# Oats Intolerance in Celiac Disease

**DOI:** 10.1371/journal.pmed.0010023

**Published:** 2004-10-19

Most patients with celiac disease can eliminate their symptoms—at a price: life-long adherence to a gluten-free diet. This means no wheat, rye, barley, and, until recently, no oats. Then some recent studies suggested that oats did not cause the intestinal inflammation characteristic of the disease, and thus oats are now often included in the celiac disease diet. This is good news for patients coping with severe restrictions on what they can and must not eat, but a study by Ludvig Sollid and colleagues in this issue of *PLoS Medicine* suggests that oats are not safe in all cases.[Fig pmed-0010023-g001]

**Figure pmed-0010023-g001:**
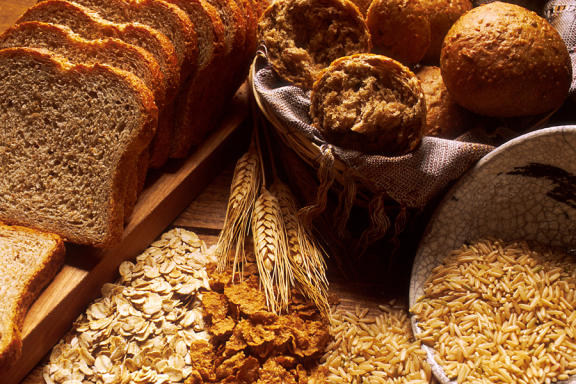
The celiac diet excludes many cereal products (Photo: National Cancer Institute)

Like other chronic inflammatory diseases, celiac disease is caused by a complex interplay between genetic and environmental factors, but it is better understood than most. Long believed to be a relatively rare disorder, it is now thought to affect about one in 250 people worldwide. Clinical symptoms are present in less than half of patients and vary considerably. Genetically, almost all patients have one of two predisposing HLA molecules, which determine the context in which their immune system encounters foreign antigens, including gluten proteins found in wheat and other cereals. In individuals with celiac disease, the immune system mounts an abnormal response to gluten, which is characterized by gluten-reactive intestinal T cells and by inflammation and compromised function of the small intestine.

Ludvig Sollid and colleagues applied the current understanding of celiac disease and a range of molecular pathology tools to studying the response to oats of nine patients with celiac disease. The nine patients were not a random sample: all of them had been eating oats, and four of them had shown clinical symptoms after oats ingestion. The goal of the study was to characterize the intestinal T cell response to oats in these patients, and to relate it to clinical symptoms and intestinal biopsy results. All patients were on a gluten-free diet and ate oats that were free of contamination by other cereals.

Three of the four patients who had reported problems after eating oats showed intestinal inflammation typical of celiac disease, and Sollid and colleagues studied intestinal T cells from these three patients. Two of the five patients who seemed to tolerate oats also had oats-reactive intestinal T cells. Functional study of these T cells showed that they were restricted to celiac-disease-associated HLA molecules and that they recognized two peptides derived from oat avenin that are very similar to peptides of gluten.

Taken together, the findings show that intolerance to oats exists at least in some patients with celiac disease, and that those patients have the same molecular reaction to oats that other patients have to wheat, barley, or rye. However, identical reactions were also seen in two of the patients who were clinically tolerant to oats. The authors suggest that these reactions could develop into symptomatic disease after some time delay, but there is no proof that the presence of oats-reactive T cells is an indicator of future symptoms or even of enhanced susceptibility to clinical oats intolerance.

Oats are not safe for all patients with celiac disease, but future studies are needed to determine the frequency of oats intolerance.

